# A Specious Plea

**Published:** 1895-02

**Authors:** 


### EDITORIAL DEPARTMENT.

F. E. DANIEL, M. D., Editor.
S. E. HUDSON, M. D., Managing Editor.
A. J. SMITH. M. D., Galveston, Associate Editor.

EDITORIAL STAFF:

PROF. J. E. THOMPSON, M. D., Texas Medical College, Galveston; Surgery.
PROF. WM. KEILLER, M. D., Texas Medical College, Galveston; Obstetrics and Gynecology.
PROF. DAVID CERNA, M. D., Texas Medical College, Galveston; Therapeutics.
PROF. A. J. SMITH, M. D., Texas Medical College, Galveston; Medicine
DR. ISADORE DYER, Tulane University; Dermatology.
DR. R. H. L. BIBB, Saltillo, Mexico; Foreign Correspondent.

Official organ of the West Texas Medical Association, the Houston District Medical Association, the Austin District Medical Society, the Galveston County Medical Society, and several others.

### A SPECIOUS PLEA.

It was the immortal "Josh Billings," we believe, who said, "the worst thing for a man to know is something that 'aint' so." A superstructure, however grand and imposing, will tumble down if built on an insecure foundation; so the most glittering deductions, drawn from incorrect premises, will fade, vanish into thin air, when it can be shown that there is no foundation in fact for the predicate; when it can be demonstrated that it is an assumption,—something taken for granted, without proof, and not a fact, the props are knocked from under the argument, and the superstructure tumbles down.

Our grave neighbor of the *Texas Sanitarian*, in the January number, has an editorial which illustrates and carries out this idea. It is very pretty, well written, and plausible; it shows up an awful condition of things which demands immediate reform. The principal trouble with it, however, is, it is built upon an assumption of something that "aint so."

The *Texas Sanitarian* has good authority for its predicate, but there certainly is a mistake somewhere, as we will endeavor to show.

Taking Dr. Jerome Cochran's paper, published in the *Alabama Medical Age* for Nov., '94, for its basis, the *Texas Sanitarian's* indignation is aroused by a statement of the author with reference to the Mobile quarantine; and assuming that the author knows whereof he speaks, takes his word for it, that the Mobile quarantine is weak and unreliable; and thereupon writes a lengthy article advocating the surrender of State control of quarantine to the United States government, and the organization of a board of health in Texas. He says:

"It will doubtless be as surprising to many of our readers as it was to us, to learn that the quarantine service at Mobile is not subject to the State Board of Health, nor of the Mobile Board of Health, but is a 'close corporation,' composed of several merchants and business men, and two physicians. The business members of this quarantine board are not quarantine experts, and possess but little, if any, scientific knowledge of quarantine matters. They are doubtless selected for these positions solely to protect Mobile's commercial interests. The two doctors on the board may or may not be quarantine experts; but be that as it may, their votes and their voices would count for little when the commercial interests of Mobile are jeopardized by a rigid enforcement of quarantine rules, notwithstanding the doctors on the board believe this necessary. As Dr. Cochran says: 'Mobile is a commercial city. Her merchants are naturally impatient with any restraint placed on her commercial intercourse with foreign countries, and so is under special temptation to relax her quarantine rules to such a degree that they could not be depended upon to afford reasonable protection to the people of the State.'"

Surprising indeed, for such is not the case by any means, with all respect to our neighbor and Dr. Cochran.

The doctor then reasons from this, taken as a fact, that all other States are endangered;—a defective predicate, and fallacious deductions, as a matter of course.

As to Mobile being a commercial city, the same can be said of all sea coast cities, New Orleans, Galveston, Charleston, etc.; and the same conflict between the commercial interests and the public safety exists there, and the same "special temptation to relax," etc.; but we will aim to show that however good authority Dr. Cochran is, and however well informed generally about quarantine matters he may be, he must be mistaken as to the Mobile quarantine being subject only to control of the business interests, and is, therefore, practically no quarantine. The argument the *Texas Sanitarian* makes, therefore, for the surrender of State control of all quarantines to the United States government, because, as asserted, the State does not control the Mobile quarantine, is no argument at all. Such a state of affai rsas is represented to exist at Mobile would not be tolerated a moment; else Surgeon-General Wyman of the Marine Hospital Service, and his inspectors, have been derelict, and ought to be impeached; for, to our certain knowledge, all the Gulf State quarantine stations have been diligently inspected by Surgeons Gassaway and Kenyoun, and others, and if there had been such a condition existing at Mobile, the Marine Hospital Service are legally obligated and required to take charge of the port, and put an officer on duty; as we will presently show.

Regarding the want of a uniform set of rules, it is a little remarkable that this usually well informed gentleman but debutant editor, is not better posted. Prior to inspection of stations, a close comparison of all State quarantine rules was made, with the rules promulgated by the Marine Hospital Service under the law of February 15, 1893, to which we will presently more fully refer, to see that there was no conflict; that they corresponded, and were uniform; and where they did not, the Marine Hospital service amended them, or made additional rules, and caused them to be enforced by State authorities, thus creating the very condition, the absence of which the *Texas Sanitarian* bemoans and makes part of his special pleading for Uncle Sam to help Texas hold down the quarantine end of our sanitary work, and calls on the Texas Legislature to give us a State Board of Health to do what is now done under the present law efficiently and satisfactorily, and actually without cost to the State; i. e., look after local outbreaks of infection in the State; for under the present law, county physicians do that, under the orders, it is true, of the State Health Officer; but they are paid by the county. The law was enacted especially to relieve the State of this burden. The doctor does not know perhaps, that prior to that enactment the State had to pay the expense of all small pox cases and all other infectious diseases,—pay for the infected articles burned, etc; and that those items amounted sometimes to many thousand dollars annually, and that at this moment, there are claims of the kind against the State, unpaid, amounting to something like sixty thousand dollars, incurred under Ross' administration, and before the present law went into effect and made each county pay the expense of its own epidemics. Hence, if we uuderstand the doctor,—to take away quarantine from the Texas health system—there would be only internal sanitation to look after, and he asks for a board to do this. We differ with him as to the advisability of such step, however strong an advocate of a State Board of Health the "Red Back" is known to be. To take away State control of quarantine would, in our judgment, render a State Board unnecessary; but be that as it may—a difference of opinion,—until the legislature can be made to see the necessity of *giving Texas a State Board of Health comprising all the functions of a State Board*—QUARANTINE, *vital statistics, supervsion of sale of foods, drugs, etc., supervision of streams to prevent contamination, a bacteriological laboratory, etc.*, something the profession has not heretofore been able to do, and we have had to accept just what they would give us,—we incline to the belief that it is best, wisest and most economical to let things rock along as they are; which we endeavored to show in our last issue was very well indeed. And here we take occasion to say, and the writer is in position to say and to know, that the head of the present health system is as strong an advocate of a board of health as any one; but like the writer, he wants a board *with all that is implied by the term* and not a make shift; and he thinks as we do, that in order to have such board, it is by no means necessary, and certainly not advisable, to give up quarantine control.

Dr. McLaughlin, the *Texas Sanitarian's* new editor, is honest and earnest in his appeal for a board; but in our judgment, goes too far when he proposes to surrender to the United States, State control of all quarantines. Here we have, first, a life-long democrat voluntarily offering to abandon the principles for which our fathers "fit, bled and died"; for, if the right to guard and protect her own borders against disease, be surrendered, why not all other State rights? Why not turn over our judiciary and legislative,—the military, the school system,—every right as a State? The principle is surrendered when one single right is conceded.

But the remarkable part of this phase of the doctor's subject is, he *asks the Texas Legislature to do it.* He calls upon them to "seriously consider whether it is not better to *transfer the management and expense* of our coast quarantine to the national government [with a big N, and a big G], in view of the danger"—imagined to exist in consequence of the (assumed) defectiveness of Mobile's quarantine!

It would, indeed, be a refreshing spectacle to see the Texas Legislature endeavoring to make the transfer! Such thing might be done by an act of Congress,—and here comes in another remarkable feature of the doctor's argument,—for as a matter of fact, Congress did, on the 15th day of February, 1893, *practically* give the United States Marine Hospital Bureau *control* of *all* quarantines, State and municipal (without the *expense*, however), and empowered the Supervising Surgeon General to set aside any quarantine officer of any State or municipal station, and put an U. S. M. H. S. Surgeon in charge at his option,—his *opinion* of the *efficiency* of any station to protect, being formed upon reports of his inspectors who, under the law, as we will presently show, are required to inspect, and who did, twice, last fall, inspect every station from Maine to California. The doctor evidently didn't know this. Where has the author of the *Texas Sanitarian's* editorial, which is as full of holes as a musquito bar, *been* all this while? What sort of a "Rip" sleep has he been in that he should not have known of facts of so much importance as the existence of the right of supervision, inspection and *control* of quarantine by the Federal Government, given the Marine Hospital Service under the law we quote below? against the passage of which all the matter of conflict between commerce and public safety was gone over in Congress for weeks,—the question of State rights talked threadbare, urged against it in vain,—and the "black eye" thought by some to have been given State rights in giving the Marine Hospital Service the right to "invade the territory of a State without an invitation." Why, the first section we quote shows how empty is the assertion that Mobile practically has no quarantine:

Section 1 of said act provides: "That it shall be unlawful for any merchant ship or other vessel from any foreign port or place to enter any port of the United States except in accordance with the provisions of this act and with such rules and regulations of State and municipal health authorities as may be *made in pursuance of*, or *consistent with*, this act." * * *

Section 3 prescribes: "*That the Supervising Surgeon General of the Marine Hospital Service shall, immediately after this act takes effect, examine the quarantine regulations of all State and municipal boards of health*, and shall, under the direction of the Secretary of the Treasury, co-operate with and aid said State and municipal boards of health in the execution and enforcement of the rules and regulations of such boards and in the execution and enforcement of the rules and regulations made by the Secretary of the Treasury to prevent the introduction of contagious or infectious diseases into the United States from foreign countries, and into one State or territory, or the District of Columbia, from another State or territory or the District of Columbia." * * *

And further that * * * "at such ports and places within the United States where quarantine regulations exist under the authority of the *State or municipality which, in the opinion of the Secretary of the Treasury, are not sufficient to prevent the introduction of such diseases into the United States, or into one State or territory, or the District of Columbia, from another State or territory*,

*or the District of Columbia, the Secretary of the Treasury shall, if in his judgment it is necessary and proper, make such additional rules and regulations as are necessary* to prevent the introduction of such diseases into the United States from foreign countries, or into one State or territory, or the District of Columbia, from another State or territory or the District of Columbia, and when said rules and regulations have been made *they shall be promulgated by the Secretary of the Treasury and enforced by the sanitary authorities of the States and municipalities, where the State* or municipal health authorities will undertake to execute and enforce them; *but if the State or municipal authorities shall fail or refuse to enforce said rules and regulations, the President shall execute and enforce the same, and adopt such measures as in his judgment shall be necessary to prevent the introduction or spread of such diseases, and may detail or appoint officers for that purpose.*'"

Section 4 provides: "*That it shall be the duty of the Supervising Surgeon-General of the Marine Hospital Service, under the direction of the Secretary of the Treasury, to perform all the duties in respect to quarantine and quarantine regulations which are provided for by this act.*"

It thus appears all through the act that the national quarantine system provided for by Congress has necessarily inspective and supervisory powers [and control] over the sanitary authorities of the State or municipality; i. e., State or local stations.—*Opinion of Asst. Attorney-General U. S., in Abstracts of Sanitary Reports U. S. M. H. Service, Washington.*

But, it seems he did know something about the right of the M. H. S. to "invade a State for health or quarantine purposes without an invitation," for, while protesting that it should *not be done*, he exultingly cites an instance in *which it was done*, in support of his argument why it should be *done all the time!* He says: "Only last year a case of yellow fever got through the quarantine at Brunswick, Ga., and died in a logging camp near the city. From there it spread into the city, and no one can say where it might have gone, had not the Federal government, through the Marine Hospital Service assumed control of matters." * * * "As soon as yellow fever broke out in Brunswick," says he, quoting Cochran, "the Marine Hospital Service pushed aside the Brunswick quarantine, put a quarantine around the city, put a quarantine on all the railroads, and established a camp of detention for refuges in transit for places of asylum."

The *facts* in the case, and we got them from the United States Government *Abstracts of Sanitary Reports*, issued by the Marine Hospital Service weekly—are these:

The captain of the bark, *Anita Berwind*, in quarantine at Brunswick, in June, 1893, developed yellow fever. Passed Assistant Surgeon Dunwoody, of the M. H. S., was sent to the assistance of the local quarantine officer, and the sick man was taken to a camp remote from Brunswick, eight miles up the little river, at the mouth of which the bark was lying in quarantine, and there in charge of Dr. Dunwoody and the State officer was quarantined. He died June 27th. No means of communicating the infection to Brunswick existed, and no case occurred in that city (nor in camp) until, for some reason not stated—the Marine Hospital Service—a month and a half later—sent a young M. H. S. surgeon, Dr. Brannan, to take charge of the State quarantine. He did so, and on the 12th of August was taken sick with yellow fever, *at the quarantine*, and was carried into or went into town,—where he died. *There was no infection in Brunswick until he introduced it*, and on the 17th of August, the fifth day of Dr. Brannan's illness, and nearly two months after the captain had died in camp up the river,—Surgeon Guiteras, of the M. H. S., telegraphed Supervising Surgeon-General Wyman, M. H. S., "Diagnosis (Brennan) confirmed; prognosis bad; *thus far it appears that the city is not infected.*"

Here, then, is illustrated *one* of the beauties of the government control of State quarantine. An unacclimated officer is sent to an infected quarantine station—which had demonstrated the efficiency of quarantine, by the bye,—takes yellow fever and is *permitted to go into the city*, where he lights up an epidemic; whereas, under State control of its own quarantine—aided, it is true, unasked, by the M. H. S.,—with an experienced State officer in charge, a case occurs and is sent to the woods and quarantined, and no other case occurs, it is "stamped out," and yet Dr. McLaughlin gives the M. H. S. credit for "pushing aside" the State quarantine, and "stamping out" an epidemic, for which the State officers were in no way responsible, and which would not have occurred in all probability if matters had not been interfered with. He uses the "facts" as he gets them from Dr. Cochran, as an argument why the United States government should take entire and permanent control of all State quarantines.

With the United States government's report of the occurrences at Brunswick before him, it is hard to understand Dr. Cochran's version as quoted above by Dr. McLaughlin. It is clearly a misstatement, and could not have been intentionally so. No yellow fever "got through the quarantine at Brunswick." The disease did not "spread from there (the camp) to the city." The M. H.

S. "assumed control of matters" long before "yellow fever broke out in Brunswick," and it did not "break out there" at all, until the M. H. S. Surgeon Brannan introduced it, nearly two months after the case at camp had died.

Dr. McLaughlin has made a good argument from bad premises, and we give him credit for his intentions, but we must differ with him, as we have done. The times are not yet ripe for a successful application to the legislature to enlarge the scope of its sanitary supervision in the interest of public health, and until it is, we will have to be content, refused as we have so often been, to let well enough alone. When times get better; when a more liberal spirit prevails in legislative halls and there is a prospect of success, the "Red Back" will not only second the demand for a State Board of Health *as is a board*, but will lead the fight,—as it has heretore done.

